# Mammographic and Sonographic Findings of Intraductal Papilloma of the Right Breast: A Case Report

**DOI:** 10.7759/cureus.37034

**Published:** 2023-04-02

**Authors:** Emily Hodge, Anicia Mirchandani, Biren Shah

**Affiliations:** 1 Medicine, Central Michigan University College of Medicine, Mount Pleasant, USA; 2 Radiology, Detroit Medical Center/Wayne State University, Detroit, USA; 3 Radiology, Detroit Medical Center/Sinai-Grace Hospital, Detroit, USA; 4 Radiology, Western Michigan University Homer Stryker M.D. School of Medicine, Kalamazoo, USA

**Keywords:** chatgpt, breast mri, breast ultrasound, mammogram, breast mass, nipple discharge, intraductal papilloma

## Abstract

Intraductal papillomas are tumors that arise in the epithelial cells of the mammary duct. Common presenting symptoms for intraductal papilloma include serous or serosanguinous nipple discharge or a palpable mass. We present a case of a 48-year-old woman who presented with spontaneous right breast nipple discharge and a palpable mass. Diagnostic imaging for the patient included mammography and ultrasound with color doppler imaging that revealed a mass at eight o’clock in the right breast at a distance of 2 cm from the nipple and that corresponded to the area of palpable concern. Percutaneous ultrasound-guided biopsy of the mass confirmed a diagnosis of intraductal papilloma. Surgical excision may be required in many cases of an intraductal papilloma due to the variety of diagnoses that can be included on the differential, the increased risk for cellular atypia, and the treatment for spontaneous nipple discharge.

## Introduction

Intraductal papillomas (IDP) are benign, proliferative lesions of the mammary duct [1,2]. Although relatively rare, intraductal papillomas are identified in approximately 1-3% of breast biopsy specimens [3] and are associated with an increased risk of developing ductal carcinoma in situ (DCIS) and invasive ductal carcinoma, with the most significant risk factor being the presence of atypia on biopsy [4]. The papillomas are divided into two classifications based on location: central or peripheral [5,6]. Central IDP is typically found in the large ducts of the subareolar region and as a single mass. These lesions are more likely to cause spontaneous nipple discharge in contrast with peripheral masses. Papillomas arising in peripheral ducts are commonly multiple lesions and rarely cause nipple discharge [5,6].

IDP can be present in women of all ages, but most commonly occurs in women 30-50 years of age [6,7]. The most common presenting symptoms of an intraductal papilloma are palpable mass and single-duct, spontaneous nipple discharge (Appendix 1). When present, spontaneous discharge is typically serous or bloody, with bloody discharge presenting due to twisting of the papilloma [4].

## Case presentation

**Figure 1:**
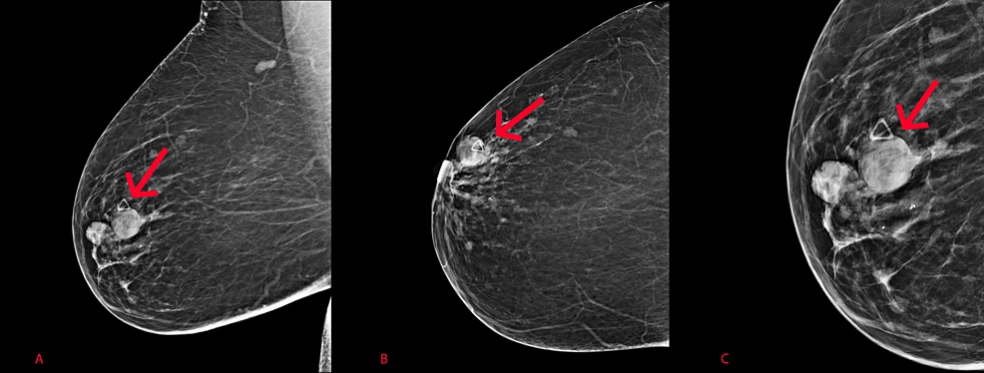
Mediolateral oblique (A) and craniocaudal (B) mammogram of the right breast demonstrating an oval, circumscribed mass located at approximately 8-9 o’clock anteriorly. Zoomed-in mediolateral oblique mammographic image (C) demonstrates the circumscribed mass (arrow) and triangular skin marker in the area of palpable concern.

A 48-year-old female presented with a palpable mass in the right breast and spontaneous clear right nipple discharge for years. The patient reported a prior history of hypertension, hyperlipidemia, and deep venous thrombosis. Pertinent family history included breast cancer in the patient’s maternal aunt. Based on the patient’s clinical presentation and exam, she was recommended for further diagnostic imaging. A bilateral diagnostic mammogram was performed and showed a high-density oval, circumscribed mass at approximately eight to nine o’clock at anterior depth (Figure 1). The left breast mammogram was negative. A triangular skin marker was placed in the area of palpable concern, which corresponded to the location of the mass seen on the diagnostic images as seen in Figure 1.

**Figure 2:**
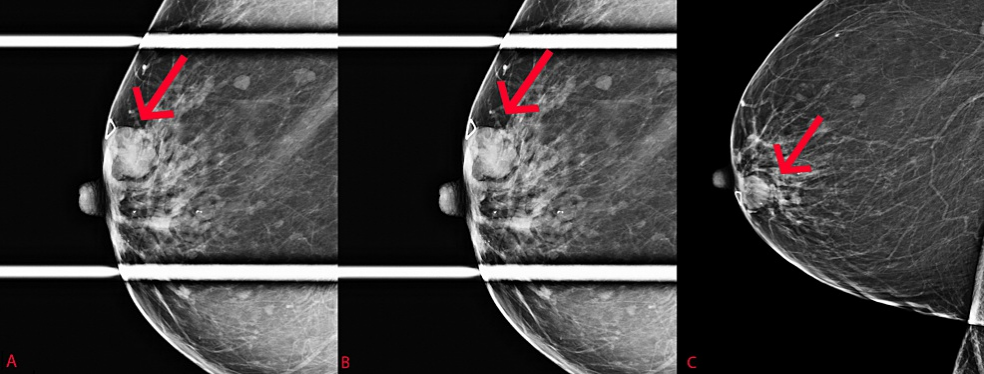
Additional right breast craniocaudal spot compression (A), mediolateral oblique spot compression (B), and lateromedial (C) mammographic views demonstrate a circumscribed mass at approximately 8-9 o’clock anteriorly, corresponding to the area of palpable concern.

Additional spot compression nonmagnification views were obtained during the diagnostic exam. These included craniocaudal spot compression, right mediolateral oblique spot compression, and a true lateromedial view of the right breast, as seen in Figure 2. This allowed for further characterization of the previously seen circumscribed mass. 

**Figure 3:**
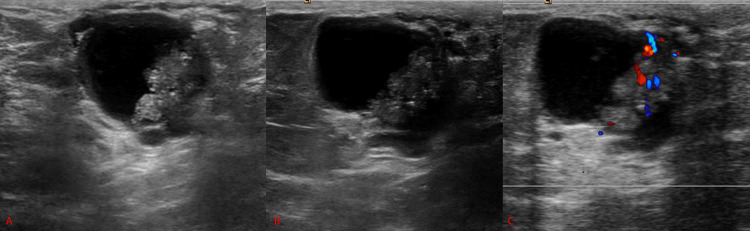
Longitudinal (A) and transverse (B) sonographic images of the right breast demonstrates a complex mass with solid and cystic components. Doppler ultrasound (C) demonstrates increased vascular flow in the solid component.

Based on the mass seen mammographically, a targeted diagnostic right breast ultrasound was performed. Ultrasound images showed a complex solid and cystic mass at eight o’clock and 2 cm from the nipple, corresponding to the mammographic mass and area of palpable concern. Color Doppler imaging shows vascularity in the solid component of the complex solid and cystic mass, as seen in Figure 3.

Based on the results of the diagnostic mammogram and targeted diagnostic right breast ultrasound, a Breast Imaging-Reporting and Data System (BI-RADS)® four category assessment was assigned. The patient subsequently underwent a percutaneous ultrasound-guided core needle biopsy of the solid component of the complex solid and cystic mass with multiple core biopsy specimens obtained. 

Pathology results revealed an intraductal papilloma with sclerosing adenosis and apocrine metaplasia. The pathology results were concordant with imaging findings.

## Discussion

An IDP can be seen in mammography, ultrasound, galactography/ductography, and magnetic resonance imaging (MRI), but not all imaging modalities are necessary (Appendix 2). However, a definitive diagnosis of an IDP can only be made via pathology. 

Mammography is typically the first imaging modality for symptomatic spontaneous single duct nipple discharge, which has many differential diagnoses including intraductal papilloma, fibrocystic changes, Paget’s disease of the breast, and intraductal carcinoma [8]. On mammography, the most usual findings of an IDP are either a negative mammogram, dilated ducts [9], and/or a round or oval circumscribed mass, with or without calcifications present [10]. 

Ultrasound is performed as part of the diagnostic imaging workup following a diagnostic mammogram. The usual ultrasound findings of an IDP are a solid hypoechoic mass within the duct or as part of a cyst (complex solid and cystic mass) [9] and when color doppler is used, a solitary papilloma will show increased vascular flow within the solid component of the mass [10, 11]. Additionally, shear wave elastography can be used to provide greater characterization because malignant lesions have higher shear wave elasticity than benign lesions [12]. Complex solid and cystic masses seen on breast ultrasound have differential considerations of atypical hyperplasia, intraductal papilloma, ductal carcinoma in situ, or papillary carcinoma. The sonographic findings cannot differentiate between the diagnoses; therefore, a biopsy is needed for an accurate diagnosis [13].

Breast MRI can also be used if there is a clinical concern of an intraductal papilloma or breast carcinoma in the setting of spontaneous nipple discharge after a negative initial workup using mammography and ultrasound. There are many imaging features of an IDP that can resemble invasive ductal carcinoma (IDC). One study by Zhu et al. determined MRI findings to assist in differentiating benign IDP from malignant IDC. Findings that support a diagnosis of IDP include a round or oval shape, and circumscribed or irregular margins, with irregular margins showing no spiculation. Conversely, IDC will show an irregular shape with spiculated or irregular margins. The study also concluded that both intraductal papilloma and invasive ductal carcinoma showed rapid initial enhancement on dynamic contrast enhancement-MRI (DCE-MRI). However, IDP showed an evolution from homogenous or heterogenous enhancement in the early phase to an increased signal intensity in the periphery during the late phase. This evolution of enhancement pattern was less likely found in IDC [1].

**Figure 4:**
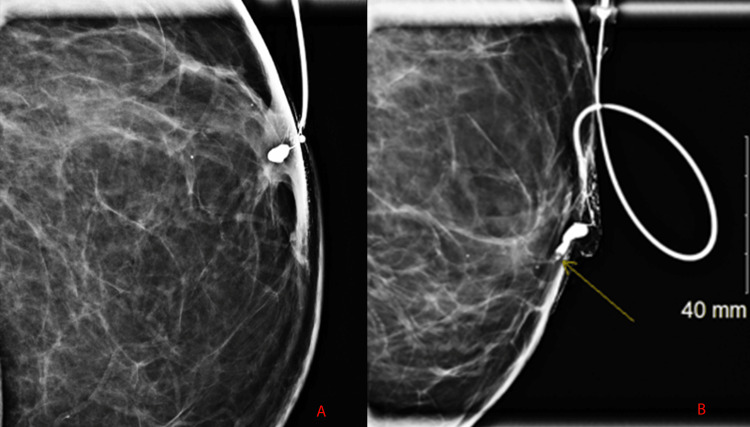
The first image, obtained from another patient, (A) shows initial contrast opacification while the second image (B) shows an abrupt filling defect immediately past the tip of the 30-gauge Jabczenski ductogram cannula.

Finally, galactography/ductography can be done to detect an intraductal papilloma. On galactography/ductography a mural-based filling defect with smooth or lobulated contours or an abrupt filling defect, as seen in Figure 4, in a different patient, from whom permission was obtained, can be seen following injection of non-ionic contrast. While a filling defect or an abrupt filling defect of an intramammary duct may represent an intraductal papilloma, other differential considerations are an intraductal papillary carcinoma, air bubble, blood clot, or inspissated material. Galactography/ductography is helpful in precisely localizing the filling defect, which can have further implications for management [14].

## Conclusions

This case demonstrated a classic presentation of intraductal papilloma with corresponding mammographic and sonographic findings representative of this diagnosis. Despite the range of imaging modalities that can be used to help further substantiate this diagnosis, a definitive diagnosis is not possible on imaging alone. Diagnosis of IDP requires either percutaneous image guided biopsy or surgical excision to direct further management and treatment.

## Figures and Tables

**Figure 5:**
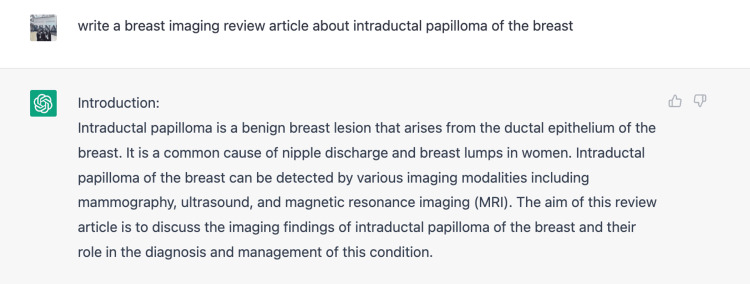
Prompt for ChatGPT to write a brief article on intraductal papilloma.

**Figure 6:**
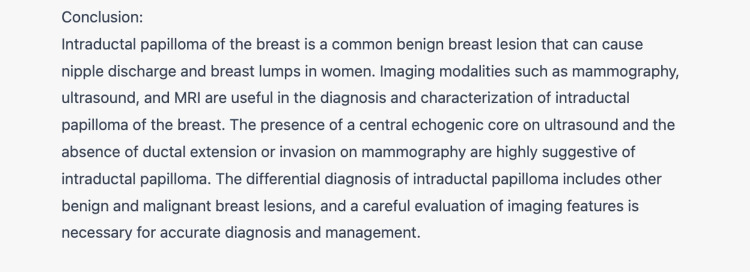
Conclusion of a brief article on intraductal papilloma written by ChatGPT that provided assistance for conceptual organization.

## Appendices

Appendix 1 

Appendix 2 
